# Current Ideas about Prebiological Compartmentalization

**DOI:** 10.3390/life5021239

**Published:** 2015-04-10

**Authors:** Pierre-Alain Monnard, Peter Walde

**Affiliations:** 1 Center for Fundamental Living Technology (FLinT), Department of Physics, Chemistry and Pharmacy, University of Southern Denmark, Campusvej 55, DK-5230 Odense, Denmark; 2 Laboratory of Polymer Chemistry, Department of Materials, ETH-Zürich, Vladimir-Prelog-Weg 5, CH-8093 Zürich, Switzerland; E-Mail: peter.walde@mat.ethz.ch

**Keywords:** protocell, compartment, lipids, fatty acids, vesicles, coacervates, amphiphiles, origin of life

## Abstract

Contemporary biological cells are highly sophisticated dynamic compartment systems which separate an internal volume from the external medium through a boundary, which controls, in complex ways, the exchange of matter and energy between the cell’s interior and the environment. Since such compartmentalization is a fundamental principle of all forms of life, scenarios have been elaborated about the emergence of prebiological compartments on early Earth, in particular about their likely structural characteristics and dynamic features. Chemical systems that consist of potentially prebiological compartments and chemical reaction networks have been designed to model pre-cellular systems. These systems are often referred to as “protocells”. Past and current protocell model systems are presented and compared. Since the prebiotic formation of cell-like compartments is directly linked to the prebiotic availability of compartment building blocks, a few aspects on the likely chemical inventory on the early Earth are also summarized.

## 1. Introduction

Whether we will ever unravel how living systems originated on Earth—or possibly somewhere else—is doubtful, as pointed out, e.g*.*, by Lazcano [1], “perhaps we will never know exactly how life originated”. Although there is no universally accepted definition of life [2], one can list at least a couple of features that are common and essential to all known forms of life. Among others, the central role of water, the ubiquity of cells as basic units of all life forms, and that of the triad DNA/RNA/protein in all life processes can be highlighted. However, and more importantly, all living systems are extremely complex with respect to their molecular components, their structure and dynamics, as well as the way they can control communication with the environment and transformations of molecules through reactions occurring within and between cellular units [3].

Due to the enormous complexity of all living entities, even in the case of seemingly “simple” prokaryotes, we have to admit that our understanding of living systems is still rather modest and superficial. Moreover, if an extrapolation is made through a phylogenomic analysis towards previously existing, now extinct, life forms, one arrives at the conclusion that even the last universal common ancestor (LUCA)—also called “urancestor” [4]—must have been already rather complex, conceptually probably not very different from today’s forms of life.

Under the assumption that living systems are nothing else than “living forms of matter” which once formed from the non-living on the basis of physical and chemical laws, there are two thoughts that are worth recalling. First, “many properties associated with living systems—such as replication, self-assembly, or catalysis—are also found in nonliving entities” [1]. Second, the origin of life problem is linked to “the origin of molecular systems having certain properties”, most significantly the capacity to confine or compartmentalize chemical reaction networks, thereby promoting reactivity [5]. Considering the prebiological emergence of a particular group of self-replicating molecules, only RNA or RNA-like compounds, is likely insufficient if one aims at developing a convincing scenario for the emergence of living cells. This has been emphasized and discussed extensively, for example by Luisi [6], Egel [7], or Koonin [8]. For instance, it has been argued that the prebiotic formation of peptides and their roles in prebiological processes—possibly together with RNA-like, as well as other molecules—should be considered more thoroughly than it has been done so far by the majority of origins-of-life researchers [4,9,10,11,12]. This reasoning leads to the inescapable conclusion that the emergence of life must be viewed as the emergence of *dynamic compartmentalized chemical systems* [5,13,14,15,16,17,18]. At first, these systems might have only performed simple tasks, but were inherently containing the “seeds” that were required to facilitate the processes leading to the emergence of the first cells. That is, early chemical systems possessed a comprehensive chemical composition or/and had the capability to acquire further complexity, although one cannot exclude that such acquisition occurred at some points by the merger of different chemical systems via processes which are comparable to symbiosis in biology.

Considering the general importance and the various roles of compartments in contemporary cells (both in terms of general cell identity and processes) [3], it is very likely that the formation of some type of compartment already occurred in early prebiotic times [4,6,9]. In this short review, the focus lies in a compilation of some of the ideas about prebiotic compartmentalization, its possible relevance for the emergence of complex reaction networks (*i.e.*, metabolism), and, by extension, for the emergence of functional cell precursor systems, so-called “protocells” [6,19,20,21,22] or “pre-cells” [23,24]. In limiting the purpose of this article, we intend to underline the likely importance of compartments in chemical systems that have been proposed for the study of the emergence of living cells. We do not aim at reviewing the whole field as other authors have done this excellently [16]. 

After a general introduction about prebiological compartmentalization and the protocell concept, we will consider some of the proposed prebiotic compartment types and the likely origins of their chemical building blocks. We will further review a few important ideas about the role which the various types of proposed compartments may have had in prebiotic times on Earth. 

Due to the extensive body of work in which the term “protocell” is used for naming artificial compartments, as micrometer- or submicrometer-sized reactor systems for possible biotechnological applications [25,26,27], we will limit ourselves to examples that are of potential prebiotic relevance. 

## 2. Models for Prebiological Compartmentalization

Compartmentalization allows reactions to occur in parallel, but in a spatially distinct manner, which is an important characteristic of all types of biological systems since the cells, as basic units of life, are themselves compartments [3]. Compartments are further involved in the efficient control of all exchanges between the environment and the cells (nutrient uptake and waste release), by harvesting and processing of energy from primary or secondary sources, as well as through sensing functions [3]. In all types of living cells, additional internal compartmentalization contributes to the function of single cells alone or in sophisticated multicellular organisms. One of the main differences between prokaryotic and eukaryotic cells is the nature of the sub-compartments boundaries, only protein [28,29] and protein/lipid [3] building blocks, respectively. 

In the field of the origins-of-life research, prebiotic compartments with some form of catalytic network are often called “protocells” [6,13,19,20,21], and are defined as hypothetical cell-like precursor systems of the first cells, which already had important features of living cells, but were not yet alive. This broad definition—without any specification of the “features” the protocell should have had—leaves room for proposing many different types of protocell model systems [6,21,30]. They range from completely inorganic structures to aggregates of macromolecules and polymolecular assemblies of amphiphiles, whereby vesicular compartments—formed from potentially prebiotic lipidic amphiphiles [6,20,31,32,33,34], peptides [10,35], inorganic nanoparticles [36,37], or polysaccharides [38]—are of particular interest since the morphology of vesicles resembles most closely that of cells. 

It would be too easy and likely wrong to limit oneself to lipidic vesicular compartments, *i.e.*, compartments with enclosed aqueous volumes (aqueous lumina), because of their inherent limitations, especially in the absence of a developed supporting protein network. For instance, the self-assembly of lipidic amphiphiles into freely floating vesicles, requires a minimal concentration of the amphiphiles (the critical vesiculation concentration, CVC) that is relatively high for prebiotically plausible molecules [39,40]. Furthermore, the formation of vesicle boundaries leads to a decreased access to chemicals from the environment, especially in the absence of evolved transport systems [40,41,42].

**Figure 1:**
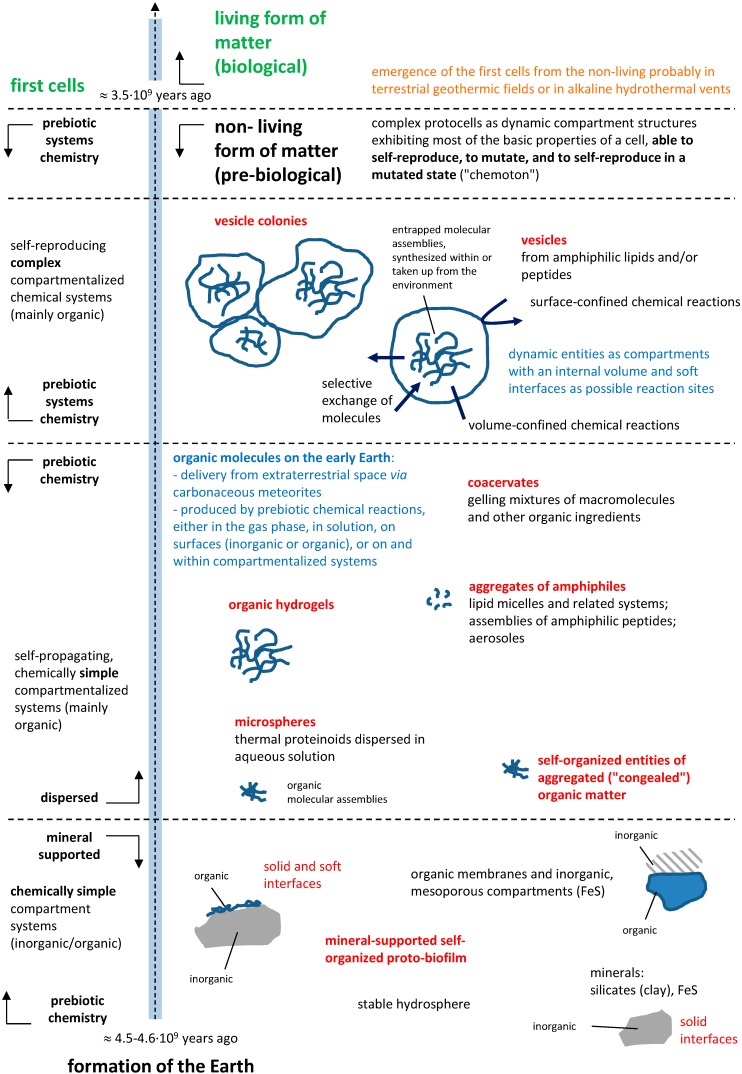
Inventory of some of the ideas about prebiological compartmentalization on Earth in a scenario that considers a terrestrial origin of life [43], see also the main text.

Figure 1 summarizes, as a kind of inventory, some of the ideas that have been discussed and some of the systems that have been investigated as possible model structures of prebiological compartments. Most popular are vesicular structures, which currently attract considerable attention as protocell compartment models [6,13,20]. In addition to vesicles with their confined aqueous volume, other even simpler compartment systems, are equally worth considering, since they may have played important roles before cell-like vesicular compartments existed, see Figure 1. Moreover, it is also reasonable to question the idea of a very early encapsulation of reaction networks into an aqueous lumen because the creation of catalytic networks or the execution of catalytic or self-assembly processes can also be facilitated by association of their molecular components with surfaces [44,45,46,47,48,49,50]. The same rationale could also apply to aggregates that possess boundaries that are not as clearly delineated as cellular membranes. In coacervates, certain chemical species can be accumulated and can perform catalysis [51]. Thus, solid surfaces and those of soft-matter structures, as well as heterogeneous phases in aqueous solutions, could have also played the roles of “pseudo-compartments” by fostering the formation of molecular concentration gradients needed to initiate catalysis. 

In the following, we will comment on and discuss Figure 1. As far as we know today, the Earth was formed about 4.5 × 10^9^–4.6 × 10^9^ years ago [52]. Over a period of several hundred million years at most, it then transformed from a hot, gaseous mass into a planet with a solid crust on its surface, which consisted of inorganic materials (rocks and minerals) and a large amount of water (about 4.35× 10^9^–3.8 × 10^9^ years ago [52,53]). The water itself may have been delivered to the Earth by impactors, e.g., chondritic meteorites [54]. It seems that the first living cells emerged from the non-living form of matter about 3.5 × 10^9^ years ago [52], although this is disputed [20]. Life already existed on Earth 2.5·× 10^9^ years ago, since microfossils from this time period have been found [20]. According to current knowledge, life on Earth probably originated in terrestrial geothermic fields [5,8,55] or in alkaline hydrothermal vents [56]. 

Contemporary biological cells are highly complex, dynamic compartment systems that are composed of organic molecules, and host networks of chemical reactions. These networks involve *organic* metabolites and catalysts that are mainly organic (proteinaceous enzymes which may contain metal ions). Thus, it appears obvious that (i) organic molecules must have already been present in prebiotic times, and (ii) the formation of organic compartments must have taken place prebiologically.

There are different ideas about the possible prebiotic inventory of organic molecules as there are about the possible structure, composition and dynamic properties of prebiological compartments. Some of these ideas are summarized in Figure 1 in order to motivate the interested reader to develop new ideas and concepts, which are highly needed if one aims at a better understanding of the origin of life. It is very likely that organic molecules—compartment-forming or not—may have been delivered to the early Earth from extraterrestrial space with carbonaceous meteorites [20]. In addition, it is conceivable that the chemical synthesis of organic molecules in prebiotic times took place in the gas phase [57], on solid surfaces (on minerals) [58,59], and/or in solution (“primordial soup” [60]). For instance, in support of a potential early role of oligo- or polyribunucleotides (“RNA-world hypothesis” [61,62]) several investigations were carried out in the past on the prebiotically plausible synthesis of nucleobases [63,64,65], nucleotides [66], oligonucleotides [46], and on the non-enzymatic replication of RNA [67]. Recently, the group of Sutherland provided experimental evidence in support of the hypothesis that precursors of ribonucleotides, of amino acids and of lipids have a common chemical origin [68]. 

Organic molecules may have self-assembled onto inorganic surfaces to form soft “proto-biofilms” [7], which may have attracted other organic molecules, which in turn facilitated intermolecular reactions due to increased local concentrations. The formation of hybrid structures consisting of mesoporous inorganic compartments (e.g., from iron sulphide, FeS) and layers of organic molecules to act as early compartment systems has been proposed [56], whereby the oxidative formation of pyrite (FeS_2_) from FeS with hydrogen sulphide (HS^−^ or H_2_S) may have been a chemical energy source [58]. Overall, these scenarios involve mineral surfaces, free or coated with organic molecules, as simple prebiotic compartment systems for localized reactions to occur.

Once, larger (longer) organic molecules were available on Earth, there must have been various ways to form dispersed compartments. These compartments would have brought other molecules into close proximity to one another, and/or have protected them by a spatial separation from the environment. Such dispersed systems may have encompassed various types of molecular assemblies, for example: “self-organized entities of congealed organic matter” [7,10]; organic hydrogels [7]; coacervates (gelling mixtures of macromolecules and other organic ingredients) [7,10]; thermal proteinoids (microspheres) [7,69]; micellar and related self-assembled aggregates of lipidic amphiphiles (including oil-in-water emulsions) [6,32,70,71]; aggregates of amphiphilic peptides [10,72] or mixtures of lipidic and peptidic amphiphiles [58]; or atmospheric aerosoles [73]. It is likely that such a compartmentalization promoted prebiotic chemical reactions via compartment-confined and surface-confined chemical reactions [74,75].

Recognizing the fact that lipid-based vesicles resemble the basic morphology of all cells, as far as the basic design is considered—an aqueous volume enclosed by a lipid-based boundary, they have been often proposed as the most cell-like models for prebiotic compartmentalization [6,20,32,72,76,77]. Vesicular compartments offer unique possibilities for mimicking the structural and dynamic properties of biological cells and their protocellular precursor structures: (i) vesicles form from potentially prebiotic lipidic amphiphiles [20]; (ii) the internal volume of vesicles can vary between about 50 nm and several 100 μm, encompassing the size range of prokaryotes; (iii) inorganic and organic molecules can be trapped inside vesicles; (iv) the permeability can be modified, or “fine-tuned”, by either choosing the membrane-forming lipids accordingly or by adding membrane-soluble compounds, or physically (e.g., by varying the temperature); (v) the vesicle interior or the vesicle surface can promote and regulate chemical reactions [50,78]; (vi) chemical reactions leading to the formation of lipidic vesicle membrane-forming amphiphiles can be accelerated by the presence of the vesicles, thereby leading to vesicle growth and reproduction [6,75,79]; (vii) vesicles can aggregate to form vesicle colonies [80]; and (viii) vesicles can entrap other vesicles to form so-called “multivesicular vesicles” [81], similar to the internal compartmentalization of eukaroytic cells. 

The most evolved protocell models towards the emergence of true cellular systems are known as “chemotons”, see Gánti [82]. Chemotons are sophisticated membrane-bound compartment systems (vesicles), which are composed of three autocatalytic subsystems, metabolism, membrane and information copying. They are “metabolizing, self-reproducing entities exhibiting most of the basic properties of a cell, but unable to limit the frequent mutual exchange of genetic information” [23,24].

## 3. Sources of Potentially Prebiotic Compartment Building Blocks 

The composition of prebiological compartment boundaries is and will remain an open question. Two possible sources can be proposed for the origin of prebiotic chemicals: (i) an exogenous one linked to the formation of chemicals in interstellar space [83] and their delivery to the Earth through impact events of meteorites [84,85,86,87] and the accretion of interstellar dust particles [88,89]; and (ii) an endogenous one linked to prebiotic chemistry on the early Earth, e.g*.*, geochemical processes [44,45,57,90,91,92,93,94,95,96]. Moreover, it seems likely that the early building blocks of prebiotic compartments were simpler than those of modern cell membranes. 

Among all possible prebiotic compartment structure-forming molecules, amphiphiles have been studied extensively [21,32,97]. Amphiphilic molecules composed of a single hydrocarbon chain with a polar headgroup, mostly a carboxylic acid function, have been generally emphasized as potentially prebiotic. However, such individual amphiphiles were probably minor components of rather complex mixtures of chemical components [90,91]. The presence of other amphiphiles with functional headgroups (phosphate, phosphonate, and amine/ammonium) can be also inferred: Traces of short alkyl chains with a functional group containing phosphorus or nitrogen [85,86,87] have been found in meteorites even though these particular molecules cannot assemble into structures by themselves. Prebiotic syntheses of phosphate amphiphiles have been proposed [33,98] that are plausible under prebiotic conditions, e.g*.*, using simple alkanols as precursors, ammonium hydrogen phosphate as reactant and urea as a catalyst under dehydrating conditions (100 °C). 

The plausibility of other potential compartment building block types has also been discussed multiple times. Since the first prebiotic synthesis by Miller and Urey [57,94], amino acids have been detected in meteorites [87] and their polymerization [99,100] and subsequent self-assembly into aggregates that could function as prebiological compartments has been studied, either as pure peptides [99,101] or in mixed systems [102]. Sugar synthesis has been known since the middle of the 19th century [95] and related derivatization processes leading to the formation of “microspherules” have been demonstrated [38]. Mineral surfaces which were proposed early on by Bernal [103] or inorganic particles formed by the weathering of rocks, e.g*.*, silicates, clays, or iron minerals, could have served as prebiological compartments [44,104,105]; they may have aggregated [106]; or they may have acted as matrices for an early prebiological compartment construction from organic molecules [47]. 

This non-exhaustive list of chemical compounds which could have been involved in the prebiological compartment formation underlines two main aspects of the chemical inventory: (i) each type of a particular chemical was represented by different species, for instance, amphiphiles with different hydrophobic tail length or types of headgroups must have been present; and (ii) various species must have been present at any given time, even some that did not directly participate to the formation of a protocellular compartment. Thus, it seems unlikely that prebiological compartments were formed by one single chemical or by one single type of chemical species only. Moreover, it is also likely that many of the chemicals or their precursors, which were important for the emergence of modern biochemistry, were involved in the spontaneous formation of prebiological compartments very early on [107]. Thus, approaches to a protocell boundary composition relying on a single pure compound, while interesting from a biophysical point-of-view to understand the physical and chemical constraints on self-assembly processes, may not allow researchers to gain a deeper insight into the emergence of cells. Although it is experimentally rather challenging, one has to try to investigate the compartmentalization of multi-component systems and not only of single-component systems. 

## 4. Experimental Approaches to Potentially Prebiological Compartmentalization 

The formation of (sub-)compartments in contemporary cells is based on chemical processes and physical interactions that enable self-assembly processes. However, the complexity of the architecture of contemporary cells clearly leads to a form of “supported” self-assembly, as the formation of organic bilayer membranes is not defined by lipids only, but also by interactions of the lipids with the cellular protein networks and with the structured intracellular medium, that is, the cytoplasm [3]. Thus, the compartment formation is ultimately determined by the genetic make-up of the cells. 

The self-assembly of potentially prebiological organic molecules into compartments has been explored under the various assumptions which reflect the constraints that could have prevailed on the early Earth. These studies can be categorized in three main groups: studies of molecules and processes which lead to compartments which are composed of (i) a single species of building blocks [108,109]; (ii) of molecule mixtures composed of a single type of building block (e.g*.*, amphiphile mixtures [40,110,111,112], coarcevates [113,114]); and (iii) more recently hybrid systems that comprise of mixtures of several types of building blocks (e.g*.*, supported amphiphile structures [47,115], coated coacervates [116,117]) or mixtures of compartment forming building blocks and other molecules [118,119]. This “evolution” of the research strategy not only reflects the desire to improve the complexity of protocell models (more stable and dynamic compartments) or to mix the properties of the building blocks and hence obtain more functional systems, but rather a desire to more accurately model the likely processes that led to the formation of increasingly complex functional compartment systems.

### 4.1. Assemblies of Lipidic Amphiphiles

Studies on the formation of compartments by a single type of molecular building blocks led to important insights into their self-assembly potential in relation with their molecular properties (hydrophobic character, solvation in aqueous medium) and environmental physical constraints (ionic strength [110], nature of counter ions [120,121], temperature [40,42] and pressure [122]). Every type of building block self-assembles once its overall concentration reaches a threshold value: in the case of plausible prebiotic amphiphiles, this concentration (so called critical aggregation concentration, CAC; CVC in the specific case of the formation of vesicles, see above) was found to be dependent on both the molecular properties (types of hydrophilic headgroups, length of the hydrophobic chains), as well as on the medium conditions. In general, for a given headgroup, e.g*.*, the COOH-group of fatty acids, the likely molecular abundance on the early Earth decreases with the increase of the hydrocarbon chain length [90]. This is simply because the formation of molecules with many C−C bonds is more difficult than the synthesis of molecules with only a few C−C bonds. Thus, plausible molecules that were present on the early Earth were most likely short fatty acids with high CACs, a fact that must have rendered the formation of protocellular structures from a pure compound more difficult [40]. Two solutions can be proposed to resolve this issue: (i) the composition of the chemical inventory was such that interactions between compartment building blocks and other species led to a decrease of the CAC as for example the study of Cape *et al.* [123] has demonstrated for fatty acid mixtures; or (ii) physical processes led to the accumulation of building blocks, e.g*.*, the association with surfaces [47,115,124]; or the differentiated flow of matter in small volumes, e.g*.*, brines in minerals [95,125]. 

Self-assembly in mixtures of single species type (e.g*.*, fatty acids and their derivatives) has been investigated as early as at the end of the 1970s [111]. The main issue for using mixtures is to establish the chemical composition of the structures in terms of effective participation of all chemicals to the structure formation. The mixtures can be categorized into two large classes: those where the amphiphiles’ hydrocarbon chain lengths were compatible (*i.e.*, of similar length), the others where the difference in carbon number is such that they may have led to the appearance of differentiated domains in the bilayers.

Mixing fatty acid amphiphiles or/and their derivatives (e.g*.*, alkyl alcohols and amines) with similar hydrocarbon chain lengths can lead to a “better” self-assembly process under a broader range of conditions. Several studies have established that such mixtures improve the stability of bilayer structures in terms of lower CAC [39,40,126], broader temperature stability [40,42,118], higher ionic strength compatibility [100,117] or most recently higher pressure tolerance [122]. In general, the improved properties can be related to stronger interactions between the head groups, e.g*.*, H-bond formation that is not pH sensitive, or the formation of ion pairs. The extent of compartment stabilization through head group interactions however decreases with increasing hydrocarbon chain length. 

Fewer studies describe attempts to investigate more complex mixtures of amphiphiles. Two possible behaviors can be observed (i) all amphiphile molecules can be detected in the aggregate structures [127] and (ii) some amphiphiles, usually short hydrocarbon chain amphiphiles, seem to enhance the propensity of longer ones to aggregate while not *per se* forming the structures [123]. 

Nonetheless, the stability of these mixed amphiphile aggregates is still relative, especially if considered in terms of effective reaction compartmentalization. Indeed, even though the compartment boundaries composed of single chain amphiphiles are more permeable than those of contemporary phospholipids, thereby allowing a more rapid access to substrates [75], the half-life of a mixed vesicle is relatively short, as demonstrated by the amphiphile bilayer mixing between two vesicle populations at temperatures as low as 20 °C [40], in contrast to that of the individual bilayers themselves. Thus, even though vesicles formed by prebiotic amphiphiles, such as small chain fatty acid (less than six carbons) and even medium chain fatty acids (more than six but less than 13 carbons), were likely involved during the emergence of the first precursor protocell systems, their involvement as closed compartments could have been limited in the absence of a supporting framework. This fact in conjunction with the realization that even the small aqueous volume enclosed in a self-assembled vesicle is still quite large and potentially unable to effectively up-concentrate all the chemicals needed to support a catalytic network has spurred a recent revival of the idea of organic coacervates and the exploration of hydrogels as compartmentalization tools for protocell models. 

### 4.2. Assemblies of Macromolecules

The exploration of coacervates, which are droplets composed of mixtures of macromolecules, as potential protocell models was pioneered by Oparin and collaborators [113] (using mixtures of gum arabic and nucleic acids). They demonstrated that these macromolecular aggregates were capable of efficiently encapsulating other large macromolecules, such as enzymes, e.g*.*, the polynucleotide phosphorylase, which can catalyzed the synthesis of single RNA strands with random nucleotide sequence in the presence of an excess of nucleotide diphosphate as monomers. Other more recent examples [128] have confirmed that similar macromolecular assemblies can harbor enzymatic activity or up-concentrate chemical species. Other related systems based on particular types of hydrogels, e.g*.*, composed of clay particles or proteins, can also self-aggregate to encapsulate functional enzymes [129]. In both types of aggregates, a crowding effect resulted from the formation of a “gel”-like scaffold around the enzymes, which is reminiscent of that observed in cells (cytoskeleton [3]). However, the maintenance of small molecule gradients could not be achieved here even that of RNA oligomers [117], whereas vesicles composed of oleic acid segregated the same RNAs for longer time periods. This distinct behavior may be related to the lack of clearly defined aggregate boundaries in the case of coacervates.

### 4.3. Mineral Surfaces and Related Inorganic Systems

Other systems have been also proposed as prebiological “compartments”, such as mineral surfaces [42,44,46,130], inorganic nanoparticle aggregates (NP aggregates) in two solvent systems (so called colloidosomes or Pickering emulsion) [34], or compartments formed by inorganic salts [36]. Mineral surfaces, such as pyrites or clays, can offer a platform for the catalysis of chemical reactions [44,46] or reaction cycles [45], the forebears of important biochemical syntheses. Related investigations showed that the adsorption of monomers, like activated nucleotides, induced their efficient polymerization [130]. However, the association of precursors and products by physisorption, and, by extension, their dissociation, depends on the physico-chemical properties of these chemicals. The association that tends to be stronger in the case of polymers could not have solely ensured a proper compartmentalization. Interestingly, certain NP aggregates can be built as to exhibit properties comparable to those expected from protocellular compartments: the nanoparticles will self-assemble into stable chemical systems that are capable of growth and division by promoting the synthesis of their building blocks from precursors or recruitment of the chemical components present in the environment [34]. Furthermore, the aqueous phases of the systems can harbor enzymatic reactions [106]. In a sense, these colloidosomes are the equivalents of the reverse-micelles (water-in-oil emulsions with amphiphiles as emulsifiers [75,131]). Structures composed of inorganic species could have also offered the opportunity for the encapsulation of chemicals. Indeed, pure inorganic species can associate into boundaries that show a reduced permeability to solutes; however the formation of vesicle-like compartments from inorganic building blocks could only be achieved by using amphiphile-derivatized inorganic compounds [33]. 

### 4.4. Mixed Compartment Systems

Clearly, all investigated systems have valuable properties for the compartmentalization of chemical species and could therefore have played a role in the emergence of chemical networks, forebears of the biochemical networks. However, it seems obvious to the proponents of the various systems that the optimal mix for primitive compartments could have only be achieved by the simultaneous presence of several types of compartment building materials. In 1988, Wächterhauser already proposed that amphiphilic structures covered, or coated, minerals in order to retain the molecules, which had been synthesized on the minerals [132]. Hanczyc *et al.* [47] demonstrated that several types of mineral surfaces, both natural and artificial ones, could induce the formation of vesicles from organic amphiphiles in order to encapsulate RNA molecules [124]. More recently, Mann and co-workers [116] proposed a coacervate system coated by oleic acid, a single hydrocarbon amphiphile, which clearly addressed the leakiness of the coacervate cores. 

This limited overview of potential compartment systems for protocells underlines the complexity of the issue and the need for more investigations, not only with respect to their self-assembly but also to potential functionalities beyond simple compartmentalization. Indeed, the complex compartment properties and functions that were essential for the emergence of cellular life may have evolved over time (see Figure 1) and/or are the products of fusions between several types of primordial systems. As Blobel proposed in 1980 [133], primitive compartments may have first only exhibited surface activity and only later evolved into an “enclosure” that resembled the cellular membranes [78]. Indeed, many examples exist in contemporary cells where the compartment structures, the bilayers, play a central role [3]. In the absence of evolved protein catalysts, the range of compartment boundary functions may have been even more extensive as shown in the work by Deamer and co-workers on lipid bilayer mesophases as biopolymerization matrices [134,135] or by Szostak and co-workers [112] on potential evolutionary advantages imparted on protocells by their boundaries.

## 5. Possible Roles of Prebiotic Compartments 

As mentioned in the previous section, the investigations of potential prebiological compartments must be also conducted in relation to their potential functions, which can only in part be extrapolated from those fulfilled by contemporary cells and cellular sub-compartments. Indeed, in the absence of the evolved cellular machinery, one should not limit oneself to simply postulating comparable functions of prebiotic compartments, since the complex contemporary membrane functions are the results of biological evolution. However, the contemporary membrane functionalities should serve as indicators for some necessary, but not at all sufficient boundary activities. The potential activity range for prebiological compartment structures can be envisioned as structural and catalytic, and is understood here in a broad sense which includes the actuations of certain phenomena, such as triggering compartment self-reproduction (see Table 1). 

**Table 1:** Proposed potential roles of prebiological compartments in comparison with those of cells.

Cellular compartments	Potentially prebiological compartments	References
Ensure cell integrity by encapsulation	Ensure some form of integrity by co-localization and encapsulation	[13,31,136]
	Define the system compositional identity and heritable traits	[137]
Support metabolism by providing an heterogeneous medium	Promote catalytic reactions	[37,48,79]
	Stabilize reaction intermediates through incorporation into compartment boundaries	[138]
Regulate exchanges with the environment by use of complex exchange systems and simple permeability	Regulate exchanges of molecules with the environment by association and simple permeability	[40,75,139,140]
Support energy uptake and conversion	Support energy uptake and conversion	[119,123,141,142]
	Trigger self-reproduction	[143]
	Impart evolutionary advantages due to its composition	[112,144,145]

### 5.1. Co-Localization *versus* Encapsulation and Its Impact on Prebiological Catalytic Abilities

**Figure 2:**
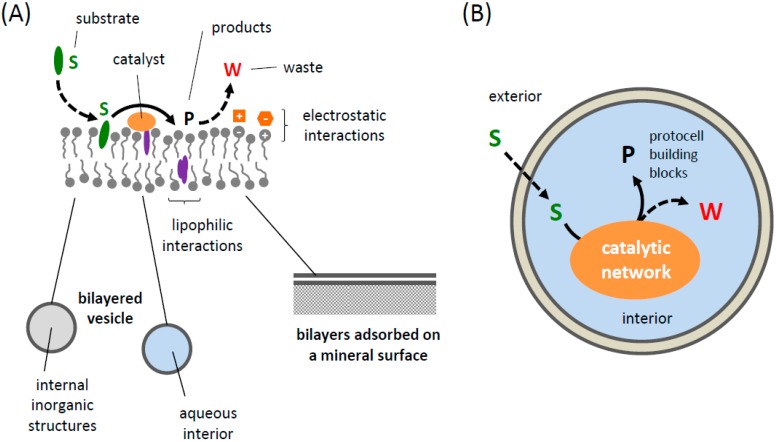
Design of protocells based on lipidic amphiphiles*.* (**A**) Interfacial bilayer systems, possibly as a boundary of a compartment system with an inorganic interior (bottom left), as a boundary of an organic vesicle with an aqueous interior (bottom middle), or adsorbed on a flat mineral surface (bottom right). The compounds that form catalytic networks can interact with the amphiphilic structure, either through a lipophilic association within the bilayers or by electrostatic interactions. In the former case, two categories of compounds exist, very hydrophobic molecules (hydrophobic chemicals or anchors are violet) that will be located within the hydrophobic core of the structures and molecules that are amphiphilic, *i.e.*, compounds with a polar or charged moiety (hydrophilic compounds or moieties are in orange) and a hydrophobic moiety that can anchor them in the bilayers. (**B**) Encapsulation of water-soluble solutes within the aqueous lumen of a vesicular compartment (shaded in light blue). In (A), a direct access to substrates (in green) present in the external environment is possible. The B design requires the substrates (S, in green) to diffuse across the bilayers either through transient packing defects or via a partitioning into the bilayers. The reaction waste (W) is in red.

The encapsulation and co-localization of protocell reaction networks in prebiological compartments likely represent the most important functions of the prebiological compartment boundary, as it is the case for the membranes of contemporary cells. One can expect that early compartments might have interacted with external molecules through associations with the compartment boundaries (in the case of amphiphile boundaries both via electrostatic or hydrophobic interactions), which may have resulted in a “trapping” of hydrophilic solutes in the aqueous lumina of the compartments (see Figure 2). However, in contrast to biological cells, the stability and permeability of prebiological compartment boundaries, *i.e.*, their effectiveness in maintaining molecular gradients, may have been paramount in defining the complexity of the reaction networks, which might have gradually evolved as the prebiological compartments became more sturdy or functional. 

The co-localization on the surface or within the compartment boundaries might have offered a more effective way to arrange simple catalytic systems than within the interior of a compartment. Indeed, the insertion of partially hydrophobic compounds, e.g*.*, hydrophobically derivatized metal complexes or other molecules [146] or polycyclic aromatic compounds, potential prebiotic photosensitizers [119,123], within amphiphile membranes or structures, like micelles [139], has been shown to allow for reactions to be efficiently carried out even leading to the reproduction of systems [48,79,143]. The advantages here would have been the relative stability of the mixed boundaries compared to that of the “neat” vesicles they form [40]. Even in the presence of chemical systems, like coacervates, the necessity of a direct association with the structure has to be postulated, considering the fact that short RNA strands can diffuse out the coacervate structures [117]. Obviously, the type of molecules that can be associated by tethering or electrostatic interactions long enough to permit catalysis is limited even though reports exist demonstrating the spontaneous inoculation of amphiphilic DNA derivatives into fatty acid vesicles [49]. Nonetheless, the potential for a co-localization on a surface of a relatively complex reaction network is plausible because essential functions on or within amphiphile compartment boundary cores have been observed: systems catalyzing transformation of precursor molecules into amphiphiles [48], or energy uptake [123,141,143]. An evolution towards encapsulation in the aqueous lumina could then have taken place as the compartment boundary composition evolved towards more stable systems composed of mixtures of amphiphiles [31,112].

Co-localization on mineral surfaces has also been envisioned [56,58]. Clays have been shown to efficiently support the non-enzymatic polymerization of activated nucleotides [46,144] and amino acids [44,147], where the association with the surface of the oligomeric products increases with increasing length. However, a too strong interaction might prevent the dissociation of the polymeric products. Moreover, the reversibility of the association by physisorption that could prevent the accumulation of small molecules, likely prompted the proposition of an amphiphile covering of catalytic mineral particles as a logical evolutionary step towards evolved protocells [132]. Indeed, the association of individual molecules into molecular assemblies, like monolayers and bilayers from amphiphiles, onto mineral particles may have led to their preservation for longer time periods as “encapsulating” molecules [124] without jeopardizing their function *per se* [115]. 

The encapsulation within an aqueous lumen—either within self-assembled structures formed from amphiphiles, such as vesicles and reverse micelles, or within porous mineral structures—cannot be excluded. The high bilayer permeability of vesicles composed of plausible amphiphiles might prevent the formation of molecular concentration gradients, while the boundaries in porous mineral structures (e.g*.*, FeS minerals) might cause the degradation of important polymeric catalysts. However, this type of compartmentalization is powerful as the expression of proteins in vesicles (*i.e.*, the encapsulation of the whole transcription/translation machinery can be achieved, yielding peptides [148] and functional proteins [149,150,151] or even allowing for the formation of chemical gradients, as the NADPH production with encapsulated TiO_2_ nanoparticles [142] demonstrated.

### 5.2. Compartment Reproduction

The capacity of cellular systems to self-reproduce is considered one of the main emergent properties of cellular systems. This phenomenon has been the subject of a number of studies with potentially prebiotic compartment systems [75,79,115,124,131,152] and also with fully synthetic, specifically designed systems [153]. The issue here is to understand how properties of an original protocell, or parent system, can be inherited by its progeny. The available studies often overlooked this aspect of self-reproduction by emphasizing the multiplication of compartments and only dealing with rather “simple systems” in terms of composition (one type of compartment building blocks). Several sources of additional building blocks have also been considered, but they are usually provided as “ready to use”. The compartment reproduction process can be decomposed into two phases: (i) a growth where a system duplicates its components; and (ii) a division phase that leads to the formation of two or more independent systems. This phenomenon presupposes relatively dynamic systems that have direct access to the building blocks (either by a direct uptake of the right chemicals or their chemical precursors followed by *in situ* chemical conversion). Most investigations on reproducing compartments pertain to organic compartments of amphiphiles [75,79,115,124,131,152]. In principle, mineral structures can reproduce using similar processes that may lead to the initial formation, as Mann and co-workers established for colloidosomes [37], or Cronin and co-workers for inorganic membranous structures [36]. In the case of coacervates, one can envision a transient disruption of amphiphile-coated coacervates due to a change in physical conditions that would lead to the formation of two similar protocell systems once the physical conditions return to the original ones. Such an idea can be postulated by analyzing the work of Keating and co-workers on heterogeneous encapsulated media [154]. All these studies establish that several potential prebiological compartments exhibit physico-chemical properties that ensure that their multiplication is achievable given the right set of environmental conditions.

### 5.3. Specific Compartment Functions

Thus far, self-reproduction processes involving potentially prebiological compartment systems have been almost solely considered from a mechanistic point of view. The unescapable issue is, however, that prebiological systems needed to have the capacity to transmit their identity to their progeny in order to first evolve towards ever more complex chemical systems and ultimately towards the first cells [13]. This question is paramount, as this type of chemical evolution would have predated the “biological” information encoded in genes that is the hallmark of contemporary cells and likely of LUCA, already. The GARD (Graded Autocatalysis Replication Domain) model hypothesis formulated by Lancet and co-worker [137] for amphiphile structures and adapted recently by Szathmary and his colleages to metabolic networks [155], attempts to answer this question. This model proposes a molecular system, called the “composome”, as the identity bearer, which could be transmitted either exactly or with slight changes to novel systems, ensuring its propagation. However, the original model only considered lipidic amphiphiles as identity defining molecules, which limits its plausibility, considering the chemical inventory and the biophysical properties of amphiphilic structures. 

Nevertheless, the idea directly pertains to a potential additional role specific to prebiogical compartment boundaries and by extension to chemical systems that preceded sophisticated protocells. Indeed, several reports exist that underline the importance of the compartment molecular make-up, but in conjunction with other aspects of the chemical systems. For example, Chen *et al.* [144] reported that osmotically swollen vesicular compartments, *i.e.*, compartments under osmotic stresses due to high solute concentrations in their aqueous lumina, will grow at the expense of other ones that are isotonic with the external medium. That is, an inherent link between an increase of internal solute concentration, e.g*.*, due to a very efficient internal catalytic activity, and boundary growth could be achieved by simply relying on physical processes, provided the amphiphile structures can maintain molecular gradients long enough. This hypothesis is supported [145] by recent observations made during the synthesis of small hydrophobic dipeptides within fatty acid vesicles, which led to the preferential growth of vesicles with a functional internal catalytic system. Moreover, the composition of compartment boundaries, themselves, can also ensure a better access to resources by increasing their stability, thus prolonging the time an internal catalytic network is preserved in a functional state [112]. Therefore, a chemical evolution by the transmission of molecule composition and their resulting catalytic properties could have already started for chemical systems (prebiological compartments) before an evolved biological information apparatus existed.

## 6. Implications of the Protocell Model Development 

Multiple designs of prebiological compartmentalization have been explored to understand the emergence of living cells on the Earth (see Figure 1). While essential insights in the potential of chemical systems were gained in these investigations, the question of the chemical nature of prebiological compartment systems remains open. The main issue is the lack of information about possible time sequences that led from the self-assembly of molecules into aggregates to the formation of complex systems that preceded the first cells, the protocells. Nonetheless, several general aspects of the problem can be outlined here. First, the multiplicity of the proposed diverse protocell make-ups can be considered advantageous for the field in general. Indeed, it is likely that various chemical systems must have co-existed during the chemical evolution leading to the first cells. That is, the appearance of protocells was not likely the consequence of a single system lineage that can be traced back to its roots on the early Earth. Second, while the investigations of individual compartment-forming molecules is useful, it seems that multi-component chemical systems, *i.e.*, complex mixtures of chemicals that together form prebiological compartments and simultaneously catalytic networks, will probably more accurately model prebiological systems. Thus, research on protocell models should emphasize its “systems chemistry” nature [13,15,16], which comes at an experimental cost: an increase of the system complexity. This idea is supported by recent developments in the fields that show the enhancement of basic functions in chemical systems that are composed of a multitude of specialized molecules. That is, the systemic bottom-up approach to the question advocated by several groups [13,14,77,156] may well be the key to unraveling the physico-chemical fundaments that support life. This statement, however, does not mean that a “correct” protocell design should encapsulate all functions that are today performed by cells. Third, to be meaningful, this approach needs to be better anchored in the correct geochemical and astrochemical backgrounds. That is, even if the use of potentially prebiotic chemicals results in less stable, less functional systems (compare the stability of contemporary lipids and their simpler counterparts, the fatty acids [157]), it is imperative that the investigated systems should intimately reflect a plausible chemical inventory. Indeed, interconnected functions of simple chemicals may help to uncover unexpected potentials for the chemical evolution of chemical systems.

## 7. Conclusions and Outlook

The emergence of functionalized prebiological compartment structures (protocells) is likely the result of a complex chemical evolution, whose sequence/stages remain(s) obscure. Although it might never be reproduced in the laboratory, the issue of the transition between inanimate and living forms of matter remains tantalizing as even a very partial answer could unlock our origins and may pave a more efficient exploration for life in space. Two obstacles clearly exist and need to be dealt with: (i) a more accurate understanding of the geochemical and geophysical conditions and their changes and (ii) a broader exploration of chemical systems in terms of their biophysics and chemical activity. The collaboration within the various communities has, thus, to be fostered and strengthened as the answer to the marvel of life does not lie in a single effort.
